# Case report laparoscopy-assisted pre-pubic urethrostomy as a palliative procedure for resection of distal urethral tumor in a female dog

**DOI:** 10.1186/s12917-021-03024-3

**Published:** 2021-09-23

**Authors:** Przemysław Prządka, Bartłomiej Liszka, Sonia Lachowska, Stanisław Dzimira, Rafał Ciaputa, Joanna Tunikowska, Łukasz Juźwiak, Paweł Kucharski, Julia Rudno-Rudzińska, Zdzisław Kiełbowicz

**Affiliations:** 1 Department and Clinic of Surgery, Faculty of Veterinary Medicine, Wroclaw University of Environmental and Life Sciences, Pl. Grunwaldzki 51, 50-366 Wroclaw, Poland; 2 Department of Pathology, Division of Pathomorphology and Veterinary Forensics, Faculty of Veterinary Medicine, Wroclaw University of Environmental and Life Sciences, C.K. Norwida 31, 50-375 Wroclaw, Poland; 3 2nd Department of General Surgery and Surgical Oncology, Wroclaw Medical University, Borowska 213, 50-556 Wrocław, Poland

**Keywords:** Surgery, urethra, cancer, endoscopic surgery, transitional cell carcinoma

## Abstract

**Background:**

This paper presents the first described case of laparoscopy-assisted prepubic urethrostomy and laparoscopic resection of a tumor of the distal part of the urethra in a female dog as a palliative treatment.

**Case presentation:**

An intact, 11 -year-old, mixed breed female dog, weighing 15 kg, was admitted with signs of urinary obstruction and difficulty with catheterization. Vaginal, rectal, and endoscopic examinations revealed a firm mass in the pelvic cavity at the level of the pelvic urethra. Ultrasound and computed tomography examination showed enlargement of the urethral wall (5.5 cm width and 3 cm thick), which was significantly restricting the patency of the urethra. The lesion affected only the distal part of the urethra without the presence of local or distant metastatic changes.

The affected portion of the urethra was laparoscopically removed while performing pre-pubic urethrostomy with laparoscopy. The patient regained full consciousness immediately after the end of anesthesia, without signs of urinary incontinence. Histopathological examination of the removed urethra revealed an oncological margin only from the side of the bladder. In the period of 2.5 months after the procedure, the owner did not notice any symptoms that could indicate a postoperative recurrence, which was diagnosed three months after the procedure.

**Conclusions:**

Pre-pubic urethrostomy can be successfully performed with the assistance of laparoscopy. The use of minimally invasive surgery will allow, in selected cases, removal of the urethral tumor, and in inoperable cases, to perform a minimally invasive palliative pre-pubic urethrostomy.

## Background

Transitional cell carcinoma (TCC) is the most common form of canine urinary tract cancer, accounting for up to 2 % of all malignancies of this species [1]. Urethral tumors are mainly described in the literature as transitional epithelial tumors and affect thousands of dogs annually around the world [2–5]. The incidence of this type of tumor constantly rises, which is an increasing challenge for veterinary clinicians [3].

Tumors of the caudal/distal urethra in bitches often do not allow for their simultaneous removal and reattachment of the cut urethra using the end-to-end technique. In such cases, pre-pubic urethrostomy, vaginourethroplasty, or conservative management is suggested [6, 7]. Regrettably, vaginourethroplasty also requires sufficient length of the urethra [6]. The most frequently mentioned methods of palliative treatment of patients with extensive tumors of the urethra, ensuring the patency of the urethra are: cystostomy [7, 8], urethral stenting [9–11], and transurethral resection [12].

Pre-pubic urethrostomy has been described in animals, both in experimental [13] and clinical studies [14]. The indications for the mentioned procedure include, amongst others, strictures of the distal urethra and neoplastic lesions [14]. The pre-pubic urethrostomy in open surgery described so far in the treatment of tumors of the distal urethra must often be performed through extensive surgical access through a midline laparotomy combined with osteotomy of the pelvic bones [6]. This leads to the need to perform a pre-pubic urethrostomy in or laterally from the laparotomy wound. This displacement of the urethra can cause urine to leak into the laparotomy wound or onto the medial surface of the limb [13, 15, 16]. The solution to this problem is to perform a pre-pubic urethrostomy in Linea alba assisted by laparoscopy, which has been successfully performed and described clinically in cats [17] and experimentally in rabbits [13].

Laparoscopic approach in the surgical treatment of tumors (including malignant) of the urinary tract has been used in human medicine for many years [18–20]. The technique is also used to remove TCC within the bladder [18–20].

The authors of this study presented the first case report with laparoscopically assisted pre-pubic urethrostomy and laparoscopic removal of the distal part of the urethra with the tumor in a female dog as a palliative treatment.

## Case presentation

An intact, 11 -year-old, mixed breed female dog, weighing 15 kg was admitted with signs of urinary obstruction and resistance to inserting a urinary catheter into the urethra. Signs of urinary obstruction had been recorded by the owner two weeks earlier. During this period, the patient was treated conservatively in another clinic, and after the clinical symptoms worsened, he was referred to the Department and Clinic of Surgery, Faculty of Veterinary Medicine of Wroclaw Environmental and Life Sciences. After admitting the patient to the clinic, vaginal and rectal examinations revealed a firm mass in the pelvic cavity at the level of the pelvic part of the urethra was perceptible. Abdominal palpation revealed a severely distended bladder. After a clinical examination and blood screening test, the patient was referred for ultrasound, CT, and endoscopy. The ultrasonographic examination revealed a strongly filled urine bladder, occupying almost the entire mid abdomen, and extending to the cranial abdomen. The ultrasound examination revealed only the initial, clearly extended (about 1.3 cm) section of the urethra, apart from the bladder. However, no cause of obstruction was visualized in the segment available for examination. The urethra and the wall of the entire bladder available for ultrasound examination did not show any hyperplastic changes.

**Fig. 1:**
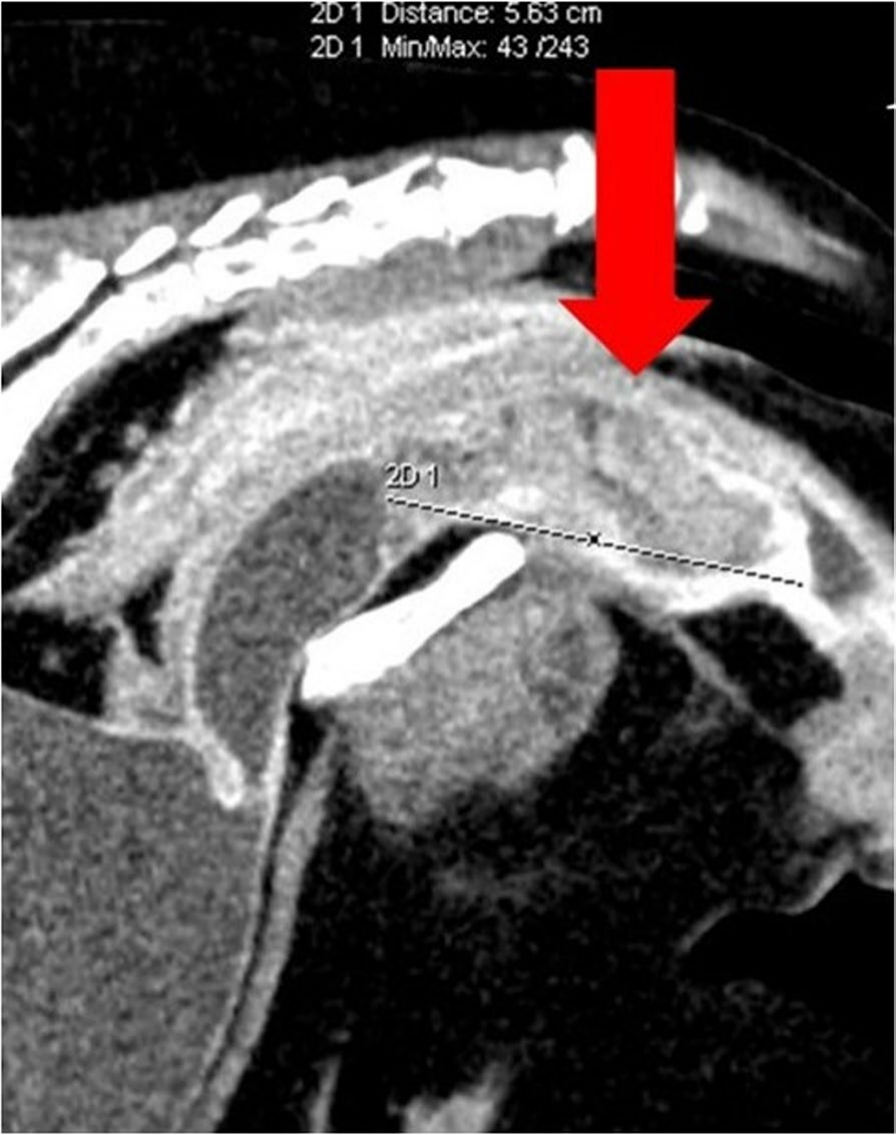
Pre-operative CT scan demonstrating the urethral tumour (red arrow)

Computed tomography examination showed well-contrasting, heterodense enlargement of the urethral wall extending into the lumen. The growth was about 5.5 cm long (taking up about 3/5 of the length of the entire urethra). The lesion was about 3 cm thick, significantly restricting the patency of the urethra (Fig. 1). The part of the urethra in front of the tumor was approximately 1.5 cm in diameter. Cranially there was a 1.3 cm long, hypodense growth coming out of the tumor into the lumen of the urethra. Contrast CT examination did not reveal any metastatic changes, both local and distant. This also applied to the bladder, lymph nodes, and lungs.

Prior to surgery, vaginoscopy and an attempt of urethrocystoscopy was performed to localize the lesion that prevents the outflow of urine from the bladder (Storz rigid endoscope, diameter of 2.7 mm). Endoscopic examination showed no macroscopic changes in the lumen of the vagina and in the external exit of the urethra. During the attempt of performing urethrocystoscopy, a mass occluding the lumen of the urethra (0.5 cm from the external exit of the urethra) was found without the possibility of introducing the optics into the lumen of the bladder. During the same examination, a fine-needle biopsy was also performed for cytological evaluation of the examined lesion.

Based on the results of the clinical trials and additional examinations (only the distal part of the urethra was changed and no metastases), the owner of the animal was presented with possible options for conservative and surgical treatment. The owner of the animal did not consent for the removal of the tumor from the pathway of the pelvic symphysis osteotomy. At the same time, he accepted less invasive procedures with the possible risk of only palliative treatment. Therefore, it was decided to perform a pre-pubic urethrostomy and an attempt of removal of the altered part of the urethra by laparoscopy.

**Fig. 2:**
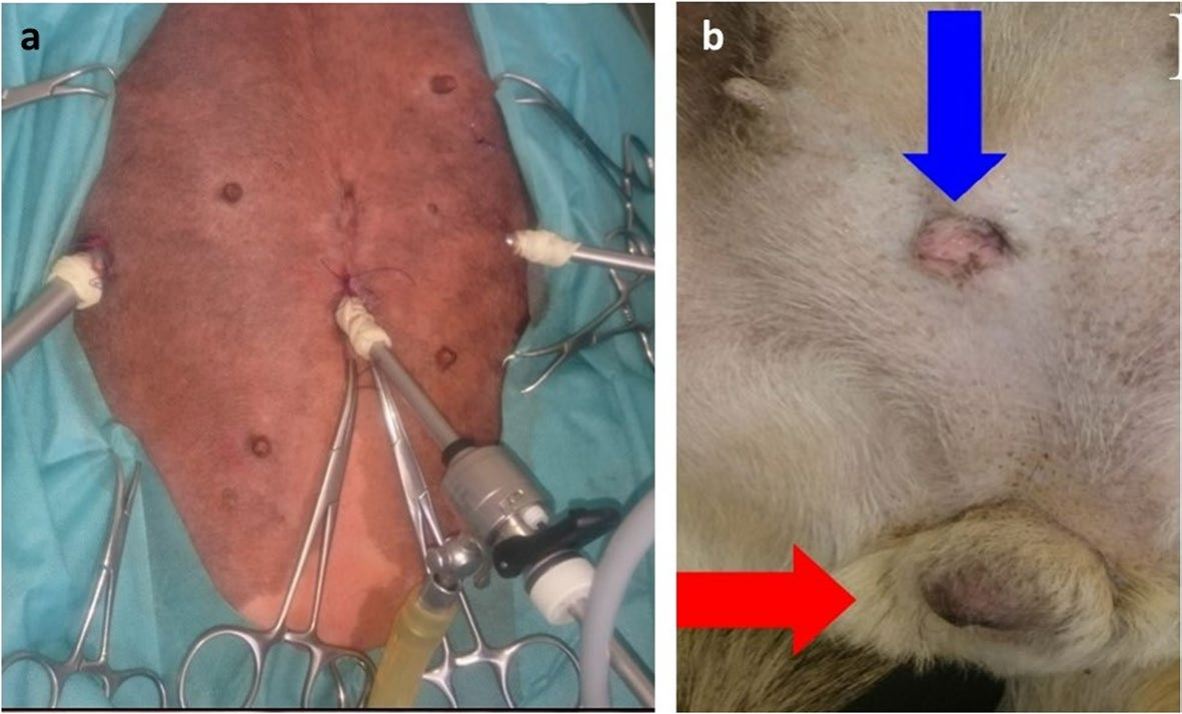
Laparoscopic pre-pubic removal of the urethral tumour demonstrating intraoperative trocar system (**a**) and post-operative urethrostomy site (blue arrow) and vulva (red arrow) at 3 months following laparoscopy

Patient was pre-medicated by intramuscular injection of medetomidine (Cepetor, CP-Pharma, Handelsges. mbH Ostlandring 13 31,303, Burgdorf Germany) at a dose of 10 µg/kg with butorphanol (Butomidor, Richter Pharma AG) at a dose of 0.1 mg/kg. Endotracheal intubation was performed after induction of general anaesthesia with propofol at a dose of 1 mg/kg intravenously to effect. After intubation, the epidural anaesthesia was provided with a lidocaine at a dose of 4 mg/kg (Warszawskie Zakłady Farmaceutyczne Polfa S.A. ul. Karolkowa 22/24; 01-207 Warsaw, Poland). General inhalation anaesthesia was maintained with isoflurane (IsoVet, Piramal Healthcare, United Kingdom) in 100 % oxygen using a circle system (Mindary Wato-Ex 65 Pro). Before placing the patient on the operating table, a catheter with a diameter of 1 mm was inserted into the bladder through which the residual urine was removed. The patient was placed in dorsal recumbency for the procedure (Trendelenburg position). After the surgical field was prepared, the procedure was started with the introduction of a 5 mm diameter optical trocar using the Hasson method in the Linea alba cranially to the umbilicus. All endoscopic equipment used for the laparoscopic procedure with a 5mm 30˚ scope manufactured by Karl Storz SE & Co. KG (Tuttlingen, Germany). After the insufflation of the abdominal cavity with an insufflator (Storz) and reaching a pressure of 8 mm Hg, optics were inserted into the abdominal cavity. Subsequently, under the control of the endoscope, two consecutive 5 mm and 10 mm diameter trocars were inserted caudo-laterally to the optical trocar in a triangular fashion (Fig. 2 A).

**Fig. 3:**
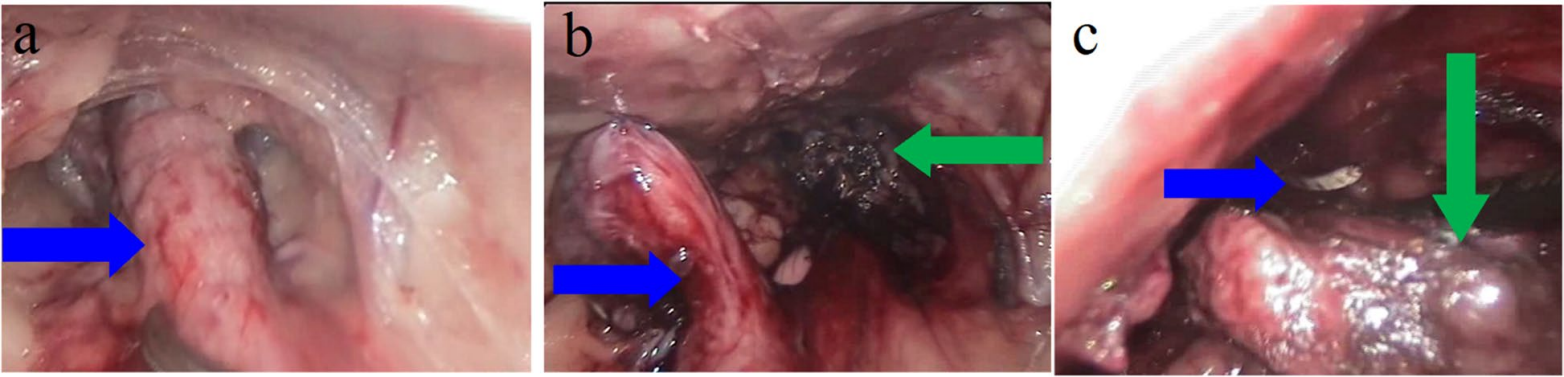
Intraoperative endoscopic imaging of the urethra. The urethra (blue arrow) as it dissects from the surrounding tissues (**a**). Following the transverse cut of the urethra (**b**), situational suture was placed closed to abdominal wall (blue arrow) with sectioning of the remaining part of the urethra (green arrow). Removal of the dissected urethra (green arrow) was made through vaginal vestibule, the blue arrow points to a haemostatic forceps inserted from the vaginal vestibule (**c**)

After accessing the abdominal cavity, the pelvic part of the urethra was successively dissected from the surrounding tissues. For this purpose, different types of vessel-sealing devices (BiCision, BiSect, Erbe Vio 3, Tübingen, Germany) were used alternately. Preparation began with cutting the median ligament of the bladder. The vesicogenital and pubovesical pouches were then opened and bluntly prepared using laparoscopic forceps. The fatty tissue present in this place, surrounding the urethra was dissected and cut off. The vessels were closed successively using the previously mentioned vessel-sealing devices. This procedure revealed a considerable length of the pelvic urethra (Fig. 3 A). Then, to improve visibility and facilitate the preparation (obtaining constant tension of the urethra without the need for an additional trocar), the urethra was suspended from the abdominal wall with a monofilament suture (Monosyn 0, Braun, Rubi, Spain). A situational suspension suture was introduced in the unchanged part of the urethra. This place was determined based on the macroscopic difference in the laparoscopic image of the dissected urethra (significant widening of the affected part). Additionally, intraoperatively, the difference in the structure (hardness) of the altered and unchanged urethra was assessed by very gentle squeezing with Maryland laparoscopic forceps along the urethra, starting from the bladder. Before cutting the urethra, the residual urine was once again removed from the bladder and the catheter was removed. A transverse urethral resection was performed using vessel-sealing devices (BiCision) in front of its thickening (affected part) and just behind the applied situational suture. After the urethra was cut transversely, behind the place of the previously conducted situational seam, the proximal part of the urethra was temporarily suspended to the abdominal wall. (Fig. 3B). The distal part of the urethra was dissected from the surrounding tissue maximally caudal up to the vaginal vestibule. This allowed for the coagulation of both cut edges of the urethra, which, combined with the earlier removal of urine from the bladder through the preoperative catheter, was aimed at limiting the possible spread of neoplastic cells. Using a speculum and per-vaginal palpating the course of this part of the procedure - initially palpable in the per-vaginal examination, movements of laparoscopic tools pressing on the wall of the vaginal vestibule, and then periodically visual inspection of cutting the opening of the urethra. After laparoscopic dissection of the urethra from the vaginal vestibule, the dissected distal part of the urethra was removed from the abdominal cavity through an opening in the wall of the vaginal vestibule using haemostatic forceps inserted from the side of the vaginal vestibule (Fig. 3 C) The surgical wound in the vaginal vestibule wall was then closed with three endoscopically inserted simple interrupted sutures from abdomen cavity. The tightness of the performed sutures was checked intraoperatively by per-vaginal examination. Then, after making a small incision in the Linea alba, the proximal part of the urethra was led out. The rim of the urethra was levelled with scissors, where the coagulated part of the urethra was removed at the time of its previous transverse cut. The urethra was sewn to the skin with simple interrupted sutures (Monosyn 4 − 0, B. Braun, Rubi, Spain). The trocar wounds were closed with simple interrupted sutures on the fascia and muscles (Monosyn 2 − 0, B. Braun, Rubi, Spain) and the same sutures on the skin and subcutaneous tissue (Dafilon 2 − 0, B. Braun, Rubi, Spain) - Fig. 2B.

After surgery, the affected distal part of the urethra was sent for histopathological evaluation, as well as the fragment of the proximal part of the urethra (from the side of the bladder) obtained during the alignment of its edges prior to the pre-pubic urethrostomy.

**Fig. 4:**
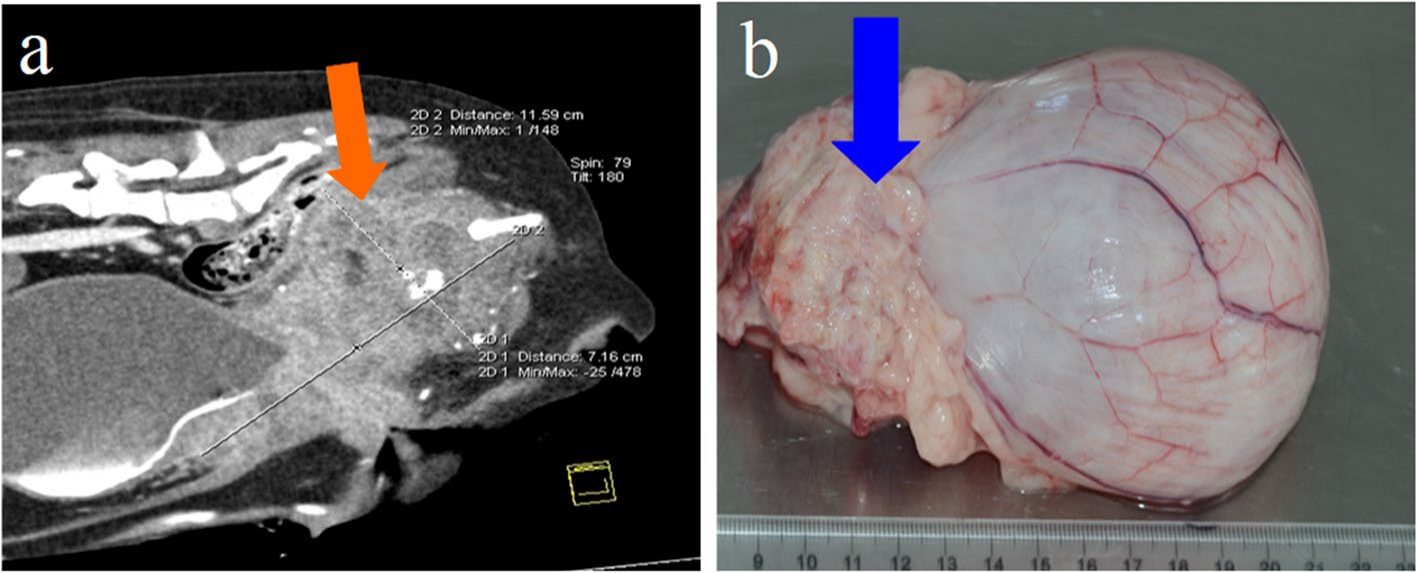
CT scan demonstrating reoccurrence of the tumour (red arrow) 3 months following surgery (**a**) with gross pathologic examination (**b**) of the tumour surrounding the urethra and bladder (blue arrow)

Immediately after recovery from anaesthesia, the patient was able to get up and move without any problems. Controlled urination, without symptoms of incontinence, was found immediately after the anaesthesia subsided. The patient was discharged from the clinic the second day after surgery. Information about the dog’s health status was transferred to the clinic by the owner by phone, where no postoperative complications were noted for a period of the first 2 weeks. After this period, the authors had no possibility of direct supervision during treatment, despite the recommendations of regular monitoring in the clinic. Lack of contact with the owner was because he lived far away from the clinic and his advanced age. Unfortunately, it was only three months after the procedure that the owner returned to the clinic with the patient showing difficulty in moving and defecating, noting while for a period of 2.5 months he did not observe any negative changes in the dog’s behaviour. Another clinical (per rectum) examination showed the presence of a firm, strongly painful mass in the ventral part of the pelvis. Subsequent CT scan showed the presence of extensive recurrence in the form of a heterogeneous tumor covering the entire width of the medial and ventral pelvic cavity and extending to the posterior abdomen, ventral and right sided to the bladder (Fig. 4 A). The enlargement completely included the pubic bones and the medial-ventral edges of the iliac bones. Additionally, metastatic changes in the lungs were visualized. The patient was euthanized, and an autopsy was performed.

A fine-needle biopsy performed before the surgery did not give a definite answer as to the nature of the examined lesion. However, the cytological specimens showed mainly erythrocytes, weakly eosinophilic, amorphous necrotic masses, few neutrophils, and single cells showing features of anisocytosis and anisokaryosis.

**Fig. 5:**
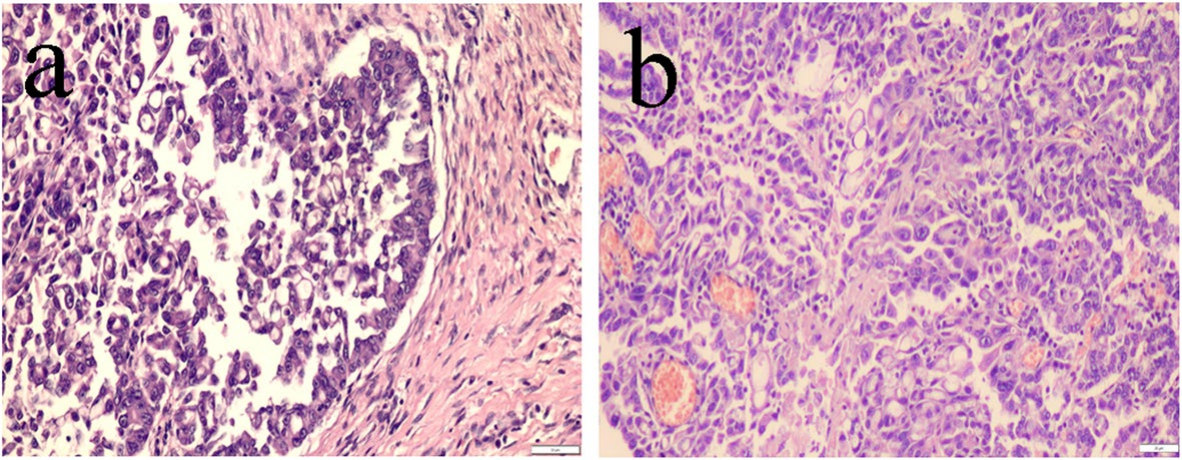
Photomicrograph demonstrating histopathological examination of the oncological margin in the incision line of the urethra from the side of the bladder (**a**) H&E, 200x. scale bar 50 μm. Invasive transitional cell carcinoma is indicated by the presence of tight nests of atypical urothelial cells with anisocytosis and anisocariosis (**b**). H&E, 400x., scale bar 20 μm

The microscopic examination of the histopathological specimens from both intraoperative specimens of the lesion, i.e., the fragment of the urethra from the side of the bladder together with the remaining removed tumor tissues, showed the weaving of a typical well-differentiated transitional cell carcinoma (carcinoma urothelial). Tumor cells were present in the incision line from the urethral opening to the vaginal vestibule. No neoplastic cells were observed in the connective tissue surrounding the urethral section in the incision line from the bladder side (Fig. 5 A). The cytological and histopathological slides were observed under Olympus BX53 microscope couplet with Olympus UC90 camera. To take acquisition the cellSens Standard V1 software was used (Olympus).

At the site of the urethrostomy, an irregular thickening of the skin and subcutis, about 1 cm thick, with no signs of neoplastic infiltration was found. The neoplastic tissue, however, comprised the urethra, circumscribed the bladder neck, and grew irregularly around these structures, reaching dimensions of approximately 12 × 7 × 7 cm. The tumor did not penetrate the lumen of the urethra and the bladder, growing exophytically (Fig. 4B). Furthermore, the surrounding soft tissues (rectal intestine and uterine body) were not infiltrated but only pressed by the mass of the tumor. The hypogastric and sublumbar lymph nodes were swollen and bloodshot. The pubic bones and ilium bones at the site adjacent to the tumor were thickened with irregular surface. Small foci of distant metastases in the form of diffuse gray-white single nodules were observed under the pulmonary pleura and in the lung parenchyma. Histopathological examination of metastatic foci revealed neoplastic cells corresponding to the image of the primary tumor - transitional cell carcinoma - Fig. 5B.

## Discussion and conclusions

According to the information available to the authors, only two papers are describing laparoscopic-assisted pre-pubic urethrostomy. Filho et al. [17] performed such a procedure on a cat with urethral obstruction. Queiroga et al. [13] evaluated the effectiveness of this technique by conducting experiments on rabbits. However, there are no similar reports in dogs. There are also no reports of laparoscopic removal of hyperplastic lesions in the distal urethra in dogs. Despite the short period of postoperative survival of the patient (amounting to three months), it is noteworthy that it is technically possible to carry out the described procedures with the use of minimally invasive surgery. It should be considered that the treatment outcome (short survival time) was due to fact that the authors did not have the possibility of long-term postoperative control of the described case and the possibility of undertaking the earlier medical intervention after obtaining the result of the presence of cancer cells in the incision line of the urethra from its exit to the vaginal vestibule. White et al. [6] found a postoperative recurrence of a transitional cell carcinoma despite the absence of metastatic changes in local lymph nodes during surgery and the preservation of a margin of healthy tissue during urethral resection. The authors of the cited study saw the disseminated nature of the growth of the above-mentioned neoplasm as the cause of recurrence, and often too later the diagnosis of this type of neoplastic changes in the urethra. At the same time, as stated by White et al. [6] surgical removal of tumors in this part of the urethra rarely gives a definitive cure, but preserves the patient’s functioning for some time. On the other hand, the authors of this study believe that the palliative result of the presented surgical procedure was probably due to the lack of a clean oncological margin of the resected urethra from the side of the vaginal diaphragm and the inability to control and continue the treatment process of the described case after the surgery.

The inability to restore the continuity of the urethra after resection of large tumors necessitates a pre-pubic urethrostomy or vaginourethroplasty to ensure urine outflow [6, 21]. The possibility of performing the latter, however, is limited by the extent of the invasive neoplastic process on the urethra, infiltration of the surrounding tissues, and the presence of local metastatic lesions [6, 21]. The pre-pubic urethrostomy procedure involves sewing the urethra before the pubic symphysis and has been described in the treatment of neoplastic lesions, injury of the distal urethra and its obstruction in males [16]. Postoperative complications include urinary incontinence, recurrent inflammation of the urinary tract, stenosis of the stitched urethra, and skin irritation around the stoma [15, 16]. White et al. [6] showed in clinical cases that after vaginourethroplasty, removal of a third of the distal part of the urethra did not cause continence disorders. The very preservation of vascularization and innervation of the proximal part of the urethra is of great importance in this matter [15, 16, 22]. In the opinion of the authors of this study, laparoscopic assistance is ideal for performing pre-pubic urethrostomy. It allows for the removal of altered organs, and in inoperable cases, to perform palliative pre-pubic urethrostomy. Additionally, the use of laparoscopy makes it possible to externalize a greater part of the intrapelvic urethra under visual control, which in the case of open surgery is partly related to the necessity to perform extensive surgical access. The surgical accesses to the urethra in open surgery include caudal laparotomy, vagina [6, 23], bilateral osteotomy of the pubic and ischial bones [6, 24, 25] and pelvic symphysis osteotomy [6, 21]. According to the authors, laparoscopic-assisted pre-pubic urethrostomy is superior to the same procedure performed by the classical method. This applies to the extent of surgical access, limited in the described clinical case to only three small wounds of the abdominal wall after trocars. The presence of an extensive surgical wound in open surgery often requires urethrostomy to be performed laterally from the laparotomy incision line, which may lead to complications such as leakage of urine on the limb, irritation and chronic dermatitis [13, 15, 16, 26]. The method presented in the study allowed for the urethrostomy to be performed at the level of the Linea alba just before the symphysis of the pubis without the risk of the above-described complications. Additionally, in open surgery, cutting the pelvic bones to dissect the pelvic urethra causes additional pain, prolongs healing and postoperative recovery. Noteworthy is the rapid return to the normal functioning of the operated patient, who moved normally immediately after the anaesthesia subsided. Comparing this with the results of open surgery in resection of urethral lesions by pelvic osteotomy, the time needed to stand up and move independently was at least 24 h after the procedure [6, 21, 25].

The disadvantage of the presented laparoscopic technique may be the relatively long procedure time of two hours, which will probably be shorter as the number of procedures performed increases. Another limitation may be the lack of appropriate laparoscopic skills by surgeons performing only basic surgical procedures endoscopically. Where, according to the authors, the most difficult stage of the procedure is the dissection of the pelvic part of the urethra with its opening to the vaginal vestibule and sewing inside the abdominal cavity. The described difficulties probably can be solved by a slight modification of the described technique by combining it with the open technique. The effectiveness of the hybrid approach requires further study on similar clinical cases. The premise of the said hybrid modification is to perform an episiotomy after laparoscopic dissection of the urethra and performing a urethrostomy will allow the dissection of the opening of the urethra and suturing the resulting wound from the vaginal side using the open surgery technique. The laparoscopic technique presented in the article allowed for the removal of the tumor in the distal part of the urethra and the performance of a pre-pubic urethrostomy. At the same time, the short period of postoperative survival of the patient indicates that the procedure performed ultimately had the character of a palliative procedure. Therefore, when choosing a method of treating similar cases (assuming that oncological margins cannot be obtained), the authors believe that other available palliative treatment methods should be considered. Among the palliative procedures enabling the outflow of urine from the bladder in the event of urethral obstruction, there are, among others, cystostomy and urethral stenting [9, 11, 27, 28]. The choice of one of the above-mentioned palliative procedures depends, among others, on a given clinical case (stage of advancement of the neoplastic process), expectations of the owner of the animal and its acceptance, possible complications accompanying a particular procedure. One study showed that the mean duration of cystostomy tube maintenance in dogs with malignant urethral lesions was 83,5 days (ranging from 1 to 363 days). Simultaneously, 49 % of animals experienced complications related to cystostomy, regardless of the indications for the mentioned procedure. Among the most frequently reported major complications related to the presence of a cystostomy tube, the following were noted: inadvertent removal of the tube or displacement from the bladder, biting of the tube by an animal, fracture of the end of the tube. Additionally, minor complications were reported, such as: irritation or inflammation around the exit side of the tube, urine leakage around the tube, haematuria, inadvertent removal of the urine collection bag, tube obstruction, bandage sores, and breakage of the suture securing the tube to the skin [28]. At the same time, another solution mentioned is urethral stenting, where the average survival time in one of the studies including the evaluation of 42 clinical cases was 78 days (range, 7 to 536). Urinary incontinence was reported in over 47 % of female dogs with a self-expanding metallic stent. In addition, the complications included recurrent urinary tract infections and urethral obstruction caused by an overgrowth of a neoplastic lesion in the urethra [11]. The patient’s survival time in the presented clinical case was 3 months, of which the period of 2.5 months included the absence of clinical symptoms visible to the owner proving the discomfort of the operated patient, such as the presence of urinary tract infections, urinary incontinence, or obstruction of the performed urethrostomy. At the same time, out of the palliative procedures mentioned above, pre-pubic urethrostomy is the most invasive, despite being performed with endoscopic surgery. Of course, the presented single clinical case cannot be the basis for comparison with the results of the works presented above and requires further research into the applicability of the surgical technique described in the paper. Especially since the authors also indicate that with the better cooperation of the dog owner (during treatment) of the described case, it was possible to perform an additional procedure from the vulva side, enabling the required oncological margin to be obtained. It should be noted that the choice of procedure in the described case was influenced by many factors, including no consent of the owner to the extensive surgical procedure, and no contact with the owner from two weeks after surgery until the diagnosis of postoperative recurrence (three months after surgery). At the same time, the authors of the study suggest that in similar clinical cases, it may be possible to combine laparoscopy with open surgery (hybrid technique), where the dissection of the urethra will be performed laparoscopically, while the pre-pubic urethrostomy of the proximal part and the final removal of the distal urethra will be performed with laparoscopic assist. Of course, in the case of non-neoplastic lesions or less aggressive changes in the urethra, the authors believe that the laparoscopic approach described in the paper may be sufficient.

The authors of this study are aware of the limitations of the objective evaluation of the presented surgical technique based on a single case, requiring further research. Especially considering the rarity of urethral tumor only in its distal part without local and distant metastatic changes present. According to the authors, the presented technique of laparoscopic removal of lesions in the distal part of the urethra may be considered, especially in the treatment of less invasive lesions of the urethra. A careful study is required for a hybrid technique, which, being a combination of the described laparoscopic technique with open surgery (an additional approach from the vaginal side - episiotomy) would lead to maintain a clean oncological margin on both ends of the resected urethra. Additionally, only palliative pre-pubic urethrostomy (as one of the palliative treatment options available) may be considered in inoperable cases.

## Data Availability

The datasets used and/or analysed during the current study are available from the corresponding author on reasonable request.

## Abbreviations

kg: Kilogram
cm: Centimetre
TCC: Transitional Cell Carcinoma
CT: Computed tomography
mm: Millimetre

## References

[1] Malfassi L, Fidanzio F, Sala M, Marcarini S, Mazza G, Carrara N (2021). A combined protocol with piroxicam, chemotherapy and whole pelvic irradiation with simultaneous boost volumetric modulated arc radiotherapy for muscle-invasive canine urinary transitional cell carcinoma: first clinical experiences. J Vet Med Sci..

[2] Mutsaers AJ, Widmer WR, Knapp DW (2003). Canine transitional cell carcinoma. J Vet Intern Med.

[3] Knapp DW, Glickman NW, DeNicola DB, Bonney PL, Lin TL, Glickman LT (2000). Naturally-occurring canine transitional cell carcinoma of the urinary bladder A relevant model of human invasive bladder cancer. Urol Oncol..

[4] Valli V, Norris A, Jacobs R, Laing E, Withrow S, Macy D (1995). Pathology of canine bladder and urethral cancer and correlation with tumour progression and survival. J Comp Pathol.

[5] Allstadt S, Lee N, Scruggs J, Bernard J, Hecht S, Callens A (2014). Transitional cell carcinoma. Vet Med.

[6] White R, Davies J, Gregory S (1996). Vaginourethroplasty for treatment of urethral obstruction in the bitch. Vet Surg.

[7] Smith J, Stone E, Gilson S (1995). Placement of a permanent cystostomy catheter to relieve urine outflow obstruction in dogs with transitional cell carcinoma. J Am Vet Med Assoc.

[8] Zhang J-T, Wang H-B, Shi J, Zhang N, Zhang S-X, Fan H-G (2010). Laparoscopy for percutaneous tube cystostomy in dogs. J Am Vet Med Assoc.

[9] Weisse C, Berent A, Todd K, Clifford C, Solomon J (2006). Evaluation of palliative stenting for management of malignant urethral obstructions in dogs. J Am Vet Med Assoc.

[10] McMillan SK, Knapp DW, Ramos-Vara JA, Bonney PL, Adams LG (2012). Outcome of urethral stent placement for management of urethral obstruction secondary to transitional cell carcinoma in dogs: 19 cases (2007–2010). J Am Vet Med Assoc.

[11] Blackburn AL, Berent AC, Weisse CW, Brown DC (2013). Evaluation of outcome following urethral stent placement for the treatment of obstructive carcinoma of the urethra in dogs: 42 cases (2004–2008). J Am Vet Med Assoc.

[12] Liptak JM, Brutscher SP, Monnet E, Dernell WS, Twedt DC, Kazmierski KJ (2004). Transurethral resection in the management of urethral and prostatic neoplasia in 6 dogs. Vet Surg.

[13] Queiroga LB, Lopes LMA, Gianotti GC, Scherer S, Alievi MM, Beck CA de C. Laparoscopic-assisted prepubic urethrostomy: experimental model in rabbit. Ciência Rural. 2018;48(1):1-6.

[14] Vives P, Braga F, Rappeti J, Milech V, Maroneze B, Möller G (2017). Prepubic urethral transposition in a male dog with extensive stenosis of the pelvic urethra. Arq Bras Med Vet Zootec.

[15] Baines S, Rennie S, White R (2001). Prepubic urethrostomy: a long-term study in 16 cats. Vet Surg.

[16] Risselada M, De Rooster H, Waelbers T, Van Geffen C, Vermote K, Kramer M (2006). A prepubic urethrostomy in a bitch after resection of the vagina and the distal part of the urethra. Vlaams Diergeneeskundig Tijdschrift.

[17] Pinto Filho STL, Oliveira MT, Souza FW, Dalmolin F, Hartmann H, Júnior ASC (2014). Laparoscopic-assisted prepubic urethrostomy in a cat with urethral stenosis. Semina: Ciências Agrárias.

[18] Patel A, Fuchs GJ (1996). Laparoscopic approaches to transitional cell carcinomas of the upper urinary tract. Semin Surg Oncol Mar-Apr.

[19] Colombo JR, Desai M, Canes D, Frota R, Haber G-P, Moinzadeh A (2008). Laparoscopic partial cystectomy for urachal and bladder cancer. Clinics.

[20] Klingler H, Lodde M, Pycha A, Remzi M, Janetschek G, Marberger M (2003). Modified laparoscopic nephroureterectomy for treatment of upper urinary tract transitional cell cancer is not associated with an increased risk of tumour recurrence. Euro Urol.

[21] Davies J, Read H (1990). Urethral tumours in dogs. J Small Anim Pract.

[22] Stone W, Bjorling D, Trostle S, Hanson P, Markel M (1997). Prepubic urethrostomy for relief of urethral obstruction in a sheep and a goat. J Am Vet Med Assoc.

[23] Tarvin G, Patnaik A, Greene R (1978). Primary urethral tumors in dogs. J Am Vet Med Assoc.

[24] Muir P, Bjorling D (1994). Ventral approach to the pelvic canal in two dogs. The Vet Rec.

[25] Allen S, Crowell W (1991). Ventral approach to the pelvic canal in the female dog. Vet Surg.

[26] Lipscomb V (2004). Surgery of the lower urinary tract in dogs: 2. Urethral surgery. In Practice.

[27] Bray JP, Doyle RS, Burton CA (2009). Minimally invasive inguinal approach for tube cystostomy. Vet Surg.

[28] Beck AL, Grierson JM, Ogden DM, Hamilton MH, Lipscomb VJ (2007). Outcome of and complications associated with tube cystostomy in dogs and cats: 76 cases (1995–2006). J Am Vet Med Assoc.

